# Returning-to-work after mental health-associated sick leave: a qualitative interview study exploring the experiences of general practitioners in Germany

**DOI:** 10.1186/s12875-023-02219-x

**Published:** 2023-12-02

**Authors:** Martina Geipel, Anna Pelizäus, Johannes Hamann

**Affiliations:** 1 Department of Psychiatry and Psychotherapy, TUM School of Medicine, Technical University of Munich, Ismaninger Straße 22, 81675 Munich, Germany; 2 Department of Psychiatry, Psychotherapy and Psychosomatic Medicine, University of Augsburg, Bezirkskrankenhaus Augsburg, Geschwister-Schönert-Straße 1, 86156 Augsburg, Germany; 3 Bezirkskrankenhaus Mainkofen, Mainkofen A 3, 94469 Deggendorf, Germany

**Keywords:** Mental health disorder, Depression, Qualitative research, General practitioner, Sick leave, Interview, Thematic analysis, Return to work

## Abstract

**Background and aim:**

Psychiatric disorders are increasing globally. Especially when these disorders affect working people, this places a financial burden on society due to long-term sick leave, the incapacity to work and the inability to earn and pay taxes. General practitioners (GPs) are often the first health professionals to be consulted by those suffering from mental health disorders.

This study investigated the experiences of GPs regarding their patients with mental health disorders and identified factors that are important for a successful return to work.

**Methods:**

This qualitative study used semi-structured interviews to explore the opinions of GPs (*n* = 12) working in Munich, Germany, or its metropolitan area. The interviews were audio-recorded, transcribed, and analyzed using the reflexive thematic analysis method.

**Results:**

GPs think of themselves as important players in the rehabilitation process of patients with mental health disorders. In their daily routine, they face many obstacles to ensure the best treatment and outcome for their patients. They also suffer from poor collaboration with other stakeholders, such as psychiatric hospitals, therapists or employers.

They indicate that the mental health disorder of each patient is unique, including the barriers to and possibilities of a successful return to work. Additionally, the workplace appears to play a crucial role in the success rate of re-entry into work. It can exacerbate the course of mental health disorders or support recovery. Fear, shame and stigmatization of the patients are personal factors responsible for prolonged sick leave.

**Conclusion:**

We conclude that GPs believe that they can have a major impact on the rehabilitation of patients with mental health disorders. As such, special focus should be placed on supporting them in this context.

**Supplementary Information:**

The online version contains supplementary material available at 10.1186/s12875-023-02219-x.

## Background and aim

Mental health disorders (MHDs) affect more than 10% of the world’s population, representing 792 million people [1]. In Germany, their 12 month prevalence is 27.7% [2]. MHDs are the second most common reason for an incapacity to work [3] and cause more days of sick leave than all other diseases [4]. The prevalence of MHDs also appears to have exhibited an upward trend over the last 15 years [4] placing a growing financial burden on public healthcare systems while also leading to huge losses in earnings. People with MHDs are also two to four times more likely to be unemployed after recovery [5].

General practitioners (GPs) are often the first health professionals to be consulted by patients with MHDs in Germany [6]. Hence, GPs are responsible for providing patients with sick leave certificates (i.e., taking them out of work) [7] and later often navigate them through the return -to -work (RTW) process [8].

Scientific literature indicates that other health professionals of the German health care system also consider GPs playing a crucial role in preventing and treating MHDs in employees [9]. Thereby it seems particularly important how the individual GP defines his or her role and how the perception of one’s role can influence the treatment and RTW process of their patients as those GPs who tend to play more roles are assumed to be more informed about their patients’ needs [10]. For example, an Australian study resulted in a broad variety of roles a GP may play when treating patients with schizophrenia, such as “ongoing management”, “family liaison and support”, and “initial crisis management” [11] where other professional groups could participate, too. There is a need for further research on the specific roles of GPs in Germany and their individual limitations, as well as an understanding of whether assigning specific roles to other health professionals might improve the treatment and RTW process of patients with MHD.

The existing international data indicate that we need to do further research on GPs themselves in order to understand their needs of improvement regarding their case management, communication skills, the impact of personal beliefs and prejudices [12]. For example, it has been shown that despite the special relationship between German GPs and their patients, the topic of future work ability is often barely touched in their conversations [13]. Little is known about the fostering and hindering factors for GPs when treating patients with MHDs in Germany, but a recently published qualitative study from Sweden could demonstrate that GPs often feel boundaries in treating patients with MHDs through the current health care system which does not provide enough time and structure to address the multifactorial needs of a patient with an MHD, whereas more communication and teamwork with other health care professionals and expanding their knowledge about individual needs of their patients with MHDs was described as a factor for care improvement [14]. Another study from France could show that most of the GPs found patients with MHDs more time- and care-consuming, more difficult to treat and needing consultations more frequently than patients with other health issues [15].

Another relevant topic is the GPs’ need of further education on work-related stress factors and MHDs, which could, according to recent studies from Germany and the Netherlands, be reached through better interdisciplinary cooperation with other stakeholders, for example occupational physicians or psychotherapists [16–18]. So far there is little information about the collaboration of GPs and other health care professionals in Germany, but data from France and Norway indicate that there is a lack of accessibility, professional exchange and collaboration between GPs and other health care professionals [19, 20]. The RTW process in Germany after long-term absence from work is commonly conducted through a gradual return between six weeks and six months, mostly initiated and accompanied by GPs [21] but in German research, little attention was paid to the strategies the GPs use to determine right time for the RTW of their patients. Data from other European countries, as well as Australia and Canada, show that the GPs experience the subject of RTW as a complex problem requiring their medical and non-medical skills and expressed their difficulties in assessing the work capacity of their patients as well as the lack of objective measures [22, 23].

Against this background, this study was undertaken to obtain a better understanding of German GPs’ practices when dealing with patients with MHDs, with a special focus on RTW issues. We wanted to learn about the factors that increase the likelihood of success in the RTW process from a GP’s perspective, as well as the obstacles that still need to be overcome to improve the rate of successful RTW. We also wanted to develop ideas about how to prevent long-term, mental health- related absences from work.

We designed six research questions (RQs) that we aimed to answer:

- 1) How do GPs describe their role in treating patients with an MHD?
- 2) What kind of strategies do they use in their everyday life with their patients with MHDs, especially when having to handle long-term sick leave?
- 3) How can their knowledge contribute to their success regarding RTW?
- 4) Where do GPs experience boundaries and difficulties with regard to treatment of MHDs and supporting RTW?
- 5) How do they perceive the cooperation with other health professionals?
- 6) What are their opinions on the established RTW methods in Germany and how should a successful RTW process look like?

## Methods

Our qualitative study followed the technique of *reflexive thematic analysis* [24, 25], meaning that this study is about the individual meanings and experiences of the GPs we interviewed [26]. Data analysis started in parallel to the data collection to determine when the information gathered by the interviews could not be broadened any further and no more new themes could be generated. We took that as a sign of data saturation and ended the recruitment.

### Recruitment and participants

Potential participants were contacted via email, phone or mail. They were chosen following the researchers’ network of contacts and using the network of K.L., who collaborated with a network of GPs as a member of the General Institute of Medicine at TUM. Participants received a short description of the study design and contact information in advance of the interviews. They were also informed that the study was performed as part of a doctoral thesis. The participants had to be trained as GPs and work at a doctor’s practice in Munich or its metropolitan area.

To broaden the focus of responses we used purposive sampling, that is, sampling of GPs from urban/rural areas and GPs of male/female gender.

Overall, 12 participants (GPs) were recruited for this study, half of whom were female. Their mean age was 57 years, the youngest being 51 years old and the oldest 70 years old. Almost all of them (11/12) worked full-time. Overall, 5 of the 12 worked at a doctors’ practice in an urban area, whereas the other seven worked in at a rural area. Most of the GPs were long-serving, having between 11 and 40 years of work experience (Table 1).

**Table 1:** Characteristics of participants

id	sex	age	part time (PT) full time (FT)	area	treatment certificates/quarter	experience/years	interview length/ min:sec	presence (P) Zoom (Z) telephone (T)
I1	f	n/a	PT	rural	n/a	18	36:52	P
I2	m	51	FT	rural	1300	19	37:57	P
I3	m	70	FT	urban	2000	30	29:38	P
I4	f	60	FT	urban	600	17	42:49	P
I5	f	52	FT	urban	530	24	25:40	P
I6	f	58	FT	urban	100	40	23:47	P
I7	m	60	FT	rural	1000	32	34:01	Z
I8	f	55	FT	rural	900	17	35:36	Z
I9	m	57	FT	rural	2500	27	34:39	T
I10	m	51	FT	urban	1600	18	45:15	Z
I11	f	53	FT	rural	900	11	40:35	P
I12	m	57	FT	rural	1200	20	26:15	T

### Interviews/data collection

Before holding the interviews, a semi-structured interview guide was created using the “SPSS” method by Helfferich [27]. The interviews took place between October 2019 and February 2021.

The first interview was performed by J.H., a male professor of psychiatry and psychotherapy, experienced in qualitative research and M.G., a female student of medicine at the time, inexperienced in qualitative research. The next eleven interviews were performed by M.G alone.

Interviews were generally held in the participants’ consultation room or office. However, some were held via Zoom or over the phone due to COVID-19 restrictions. The participants were informed that the interview was to be audio-recorded and transcribed. A time limit of 60 min, which had been discussed beforehand with the participants, was set. Each interview was held in a semi-structured form using an interview guide and ended with the completion of a short questionnaire to collect auxiliary information, such as age, years of work experience, and the workload at the doctor’s office. Each interview started with four open-ended key questions that were followed by more specific questions. This helped to avoid closed questions, or at least helped to set them aside until the end of the interview.

The four key questions covered 1) the GP’s experience regarding employed patients with MHDs in general, 2) their strategies when encountering a patient with an MHD asking for a sick leave certificate, 3) how they handled patients with long-term sick leave or patients who were in need of or returned from psychiatric inpatient treatment, and 4) their opinions on the established RTW methods in Germany and how a successful RTW process should look.

When ending the interview, the participants were given the possibility to emphasize on or add certain topics that seemed important to them. Throughout the interview, the researcher attempted to develop and sustain a lively conversation.

There were no repeat interviews carried out.

### Data analysis

Transcription was carried out following the rules of Dresing and Pehl as described in their handbook [28]. All interviews were strictly anonymized by the researchers. No transcripts were returned to the participants for comment or correction. The* Reflexive thematic analysis * [24, 25] was applied to the data, following the authors’ guide of analytic stages. It was delivered with an underlying constructionist epistemology, following an experimental orientation, meaning that this study is about the individual meanings and experiences of the GPs who were interviewed [26]. D.B., an experienced qualitative researcher, answered the questions of M.G. regarding the execution of the analysis method.

M.G. revisited all transcripts mindfully and critically while taking notes of certain items of potential importance.

Coding was done inductively, using MaxQDA software. Therefore, the whole dataset was re-read thoroughly to create codes. Some codes consisted of a few words, while others consisted of many lines of text. Each code had to be easily understandable, even without the attached data. After having completed the coding, the collected codes were revisited, sometimes consolidating similar codes or adjusting codes to be more specific. In the end, there were 955 codes in total.

As a next step, the main themes were identified. Subsequently, a search for similarities between certain data was conducted, looking for recurring ideas throughout.

This process resulted in *overarching themes*, *themes,* and *subthemes* [24]. Overarching themes and subthemes were visualized on a thematic map.

Finally, before writing the report, each theme was given a precise definition to avoid overlapping themes.

Meetings to introduce and discuss the findings with another inexperienced female researcher and at the time student of medicine, A.P., and an experienced qualitative researcher, D.B., as well as J.H., took place after the coding process and after having established the first overarching themes. K.L., a male professor at the General Institute of Medicine at TUM, offered his feedback regarding paper-writing. A.P. also helped with proof-reading, revised the written report and gave her constructive feedback.

After writing the report, we checked whether any important information was missing using the Consolidated Criteria for Reporting Qualitative Research checklist (COREQ) [29].

## Results

**Fig. 1:**
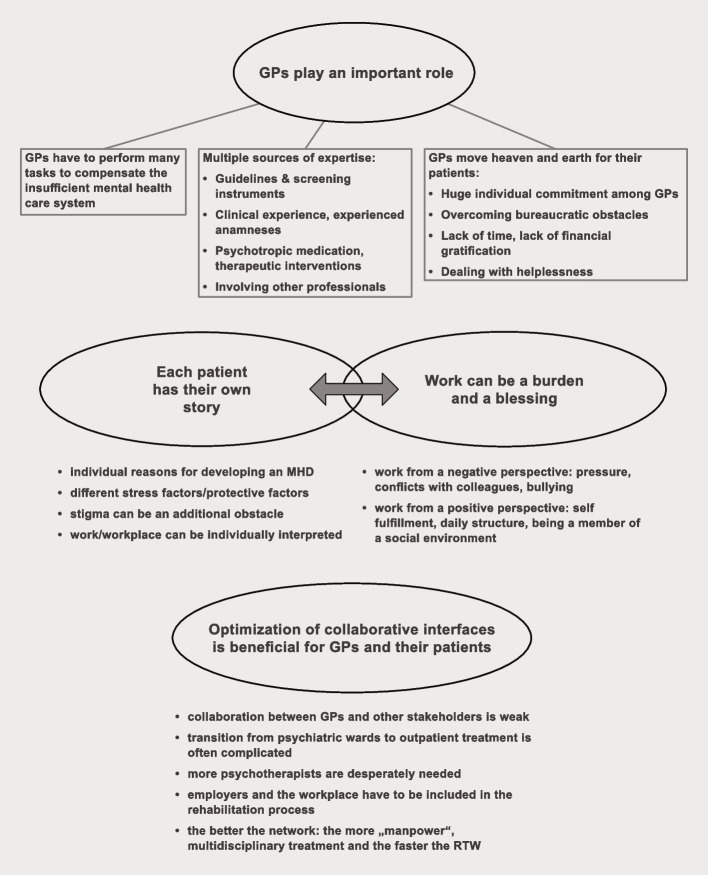
Visual map of overarching themes and subthemes

The following main themes were identified:

- 1. GPs play an important role in treating and rehabilitating those with MHDs.
- 2. Individuality is the key:
- 2a Every patient has his/her own story
- 2b Work can be a burden and a blessing
- 3. Optimization of professional (psychiatrists, therapists, hospitals) and non-professional (employers, consultants) interfaces for collaboration is beneficial for GPs and their patients (Fig. 1).

### GPs play an important role and can adopt very different roles

First, the GPs reported taking a significant part in treating and rehabilitating those with MHDs. Addressing RQ 1, various roles were described by the GPs, which can be adopted either separately or simultaneously, just as needed and requested.

#### “Counterpart, coordinator, sick certifier” (I1:35): GPs adopt various roles

**Fig. 2:**
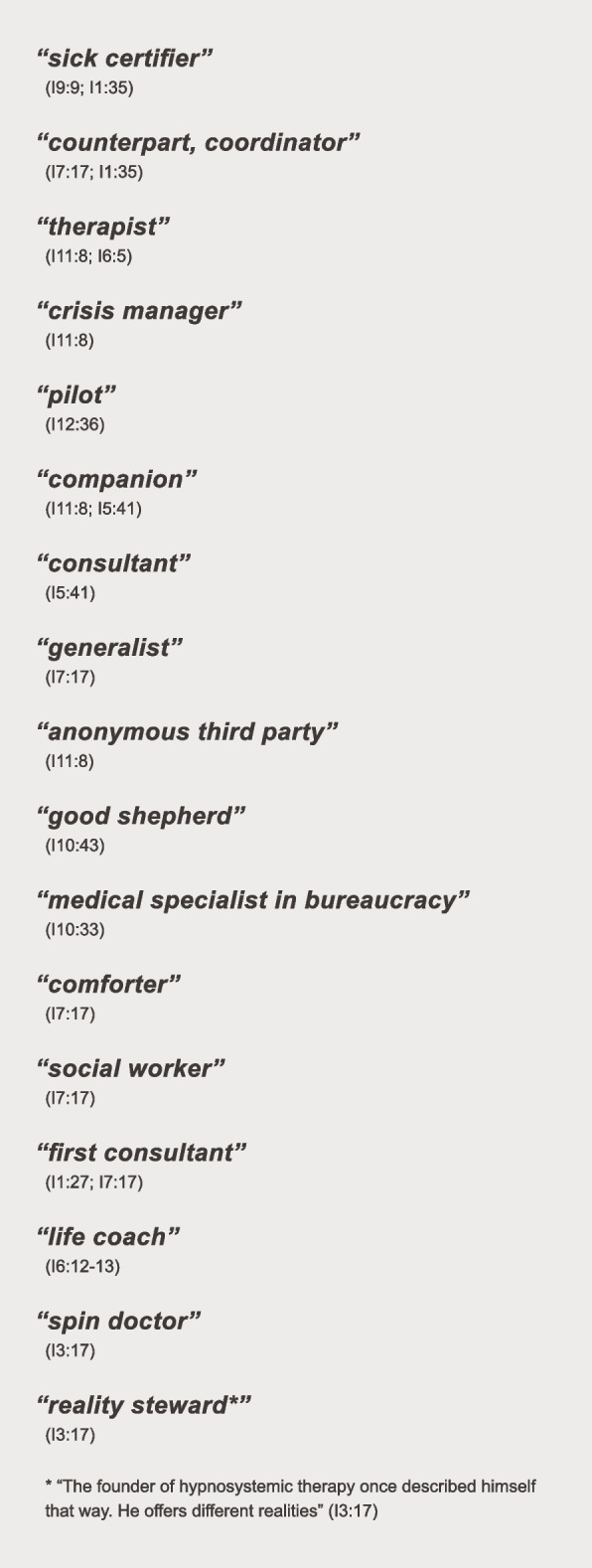
The different roles of a GP

It became clear that GPs can play the roles of different stakeholders, as shown in Fig. 2. Of course, it is important that GPs deliver a medical diagnosis and therapy options to their patients. However, we also discovered several other roles of GPs, for example, being a “generalist” or a “coordinator” to their patients. When consulting a GP, people are in need of a “crisis manager,” a “companion,” and sometimes a “comforter” (Fig. 2).

Another role is being a therapist for their patients. To compensate for the lack of psychotherapists, some GPs offer short but regular therapeutic interventions. When looking at the different roles at Fig. 2, there are some included which one might rather attribute to other non-professional and professional groups. “Comforter” for example, could also be the role of a friend or family member, “good shepherd” is reminiscent of a spiritual person (such as a priest for example), “social worker” even contains the professional group in the definition of the role.

“And then I start with conversations and then something that is basically some kind of psychological exploration. I do not do it in 1 h, I divide it up and tackle one topic each time” (I8:10).

“(…) I bypass the time, until that patient can start with psychotherapy…and we do intensified talk therapy, over a period of 2,3 weeks, while the patient is typically certified unfit for work” (I12:24).

Regarding RQ2, GPs usually come up with a treatment plan for the upcoming weeks. They reassess the patient at least once a week when being their only professional contact. Additionally, their patients obtain some type of homework in the meantime, such as visiting a friend, performing activities they enjoy, or practicing certain relaxation techniques. GPs recognize when and how to get other professionals involved, such as psychiatrists, staff at psychiatric hospitals, company medical officers, or other professional and non-professional participants.

They can also provide guidance for their patients about handling the different bureaucratic obstacles that they eventually will have to face during their sick leave.

“And yes, I also hint at them that if it will last for a longer time, that the thing with the sickness allowance is taking place. On the other hand, that the medical service will get involved” (I12:34).

GPs are often “sick certifiers” to their patients (Fig. 2). The sick leave certificate is undoubtedly one of the most important subjects dealt with in a GP’s office. The data show two types of approaches regarding the duration for which it applies: One is a short-term sick leave. It is seen as reasonable to facilitate recovery from temporary stress, often serving as the most important remedy for patients with mild forms of depression.

The other type, where patients are freed from work for several weeks or months, is far more complicated for the GPs. They feel pressured when deciding on issuing a sick leave certificate, especially when it is for a long period. They have to justify this to the health insurance system and fear that their practice will become known in town as the “sick -leave practice.” Meanwhile, they encounter patients who are reluctant to go on sick leave, even if they are already too exhausted to work. The GPs reported that some patients fear admitting to the fact that they have an MHD and hinted at the stigma of MHDs at their workplace.

“So, there are practices where you get the sick leave certificate at the push of a button. Not at our practice. We are rather restrictive in certifying [people as] unfit for work. And we rather encourage our patients to accept a sick leave certificate and take some time off” (I12:46).

“(…) I often tell the patients, especially the ones who reject being certified as unfit for work, that that is my medication. Without the side effects” (I4:28).

“But it is on the one hand the workload, how do you deal with your position at work, and how big is the fear of losing your job (…). And most of the time it does not get to them when I tell them: ‘If you would be the first one, you would be ready to be sick. But if you are the last one, who has to do the work of five, then you are the one who keeps on going, regardless. Until you drop. You must be taken into hospital first. Then, it is allowed. That is absolutely ‘schizophrenic’” (I4:30).

Additionally, the data indicate that GPs often determine the choice of therapy instead of letting their patients decide, simply because their patients are not able to do so. However, they rejected the idea that they adopted paternalistic approaches. They take these matters into their own hands because they *have to.* That role was described as being a “pilot” (Fig. 2).

“And I recognize that many people are not at all capable of managing it. Yes, and I tell them then: ‘So look, now we have figured out the disease and now we do this and that and then do that and then you will be ‘fine’” (I6:5).

“The pilot is maybe carrying things too far. Because the pilot is the one accepting the responsibilities of a captain. I am never the captain. I am the companion” (I3:15).

#### GPs need and have multiple competencies

Answering to our RQ3, GPs mentioned guidelines and screening instruments, such as the Patient Health Questionnaire (PHQ), as a source of knowledge. They help with orientation in establishing a diagnosis, monitoring the progress of their patients. and deciding on a suitable therapy.

However, clinical experience and in particular the “experienced anamneses” appeared to be even more important, meaning that GPs can witness the medical history of a patient while it is happening. They often know their patients for quite a long time when being confided in about an MHD. This leads to a clinical gut feeling, supporting them in finding the underlying cause of things more quickly. It also helps them to better identify the patients’ individual stress factors and offer solutions to them.

“And then you realize relatively fast/. So, when you work in an office for a long time, you realize rather quickly when this and that is not feeling right. That there must be something more to it. I have known many of my patients for a longer period. And, eh, that makes it easier for me, to think beyond those lines as well. To understand the background” (I2:5).

“(…) and maybe they come with some kind of stomachache or headache or simply saying: ‘I cannot do it anymore, I am so exhausted. I cannot go up the stairs anymore.’. So, you have a man who is 40 years of age, actually an athletic man. And you have the feeling at first glance that he is not consulting with you because of a somatic problem but because of something, eh, a mental problem at his workplace” (I7:21).

The topic of psychotropic medication generated very diverse reactions among the GPs. Some of them expressed a rather skeptical opinion on them. Another group suggested that they prefer to have a psychiatrist prescribe psychotropic medication instead. The third group even actively attempted to convince their patients that antidepressants and other psychotropic drugs could relieve their symptoms and thereby represent an important pillar of their therapy.

“Especially because I am someone with a holistic approach to medicine not 100% convinced that psychotropic drugs are always (…) the non-plus ultra. And, eh, that is why I think I rather stick to a path without medication as long as possible” (I4:6).

“And yes, most people tell me: ‘I do not want to take medications at all for now and, to begin with, I want to try it without them, doing psychotherapy instead. Is it necessary?’ So, I try to make it clear to them that normally you reach your goal faster with medication concerning depression, for example. Some people can be convinced that way (…) sometimes we have to do a lot of convincing there” (I10:2).

Finally, there are many administrative competencies that the GPs have to acquire, such as how to organize an RTW process correctly and how to deal with health insurance paperwork when treating a patient with an MHD. It became clear that the GPs have a big store of knowledge, but also have to develop individual competencies to handle their different roles successfully.

#### GPs move heaven and earth for their patients

We discovered a huge individual commitment of GPs when treating their patients, even if they must overcome many obstacles. Learning about them was the aim of RQ4.

For example, they often described having difficulties concerning their work environment – be it having to process the numerous forms, reports and requests by the health insurance system or the constant shortage of time. They also felt that the financial rewards for their extra work was not at all adequate.

Another surprising finding of this study is that some GPs often felt helpless when treating their patients; this led to them feeling like an imposter. This can be based on the lack of knowledge about the treatment of MHDs, especially when they are severe, the fact that there are often no simple remedies to MHDs, or even the fear of making mistakes. Their frustration and helplessness are rooted in the conviction that a GP must always have a satisfactory solution for their patients.

“(…) I find it a shame that we as doctors (…) only put on a psychosomatic coat. Eh, we get that certificate [of an additional psychosomatic education] and then never show up in the Balint group again. And, in fact, we have no clue how to deal with those kinds of things” (I3:29).

“(…) So that is…making me feel helpless, you know? Totally helpless” (I4:6).

“(…) I have the feeling of having no chance to support them when they are in an emergency. I feel completely helpless. I do not know where this is coming from (…) But it scares me. And the fear is simply from the helplessness” (I4:12).

Owing to the unsatisfactory conditions concerning the network of mental health care, GPs are increasingly required to compensate for this gap. Nevertheless, GPs try to make things work for their patients.

### Individuality is the key

We found two other important aspects that influence each other and can partly be seen as answers to RQ4.

#### Every patient has his/her own story

First, there are various MHDs, as well as many triggers for developing them. Some patients suffer a devastating event, such as the loss of a family member, while others cannot identify reasons for feeling ill. The GPs’ patients usually have multiple underlying stress factors, making it even harder for the GPs to treat their MHDs sustainably. Meanwhile, a strong social network was often described as a protective factor, as well as serving as a resource when already sick.

“(…) Generally speaking, being strongly integrated makes it easier. But I think it is mostly or rather people who have problems at work who are also not so socially integrated. And it is also those who are more vulnerable to it. So, if I have a good circle of friends and family, then I am also able to tolerate much more. Or I can take the appropriate measures more easily. So, the lonelier a patient is, the more the problems he or she has to face, in my opinion. And that is our society, too. Through mobility, our core family is often located far away. Or you are single anyway. And that is so to speak the underbelly of it” (I12:56).

Some GPs even indicate that certain personality traits aggravate the healing process. The fear of stigmatization seems to be another problem faced on the road to recovery. For example, out of shame and fear of their colleagues’ reactions, patients cannot open up about their disease at work.

“Working patients having a mental disorder (…) to begin with, they have the problem of not being able to be open about it. Eh, because I think our society is still very dishonest. In the media, we are shown mental illness as being normal. Celebrities have depression, celebrities have bipolar disorder, have borderline [personality disorder] in their youth. In reality, mental health issues are still handled differently than somatic diseases. And the stigma is definitely ‘greater’” (I11:2).

Especially if the circumstances in the workplace contributed to the development of disease, the workplace will likely also influence the prognosis of a successful RTW.

#### Work can be a burden and a blessing

Work can be either the fuel to an MHD or part of its cure. Increasing work, conflicts with colleagues, or even bullying can often lead to a mental breakdown. The GPs described a negatively interpreted workplace as one of the main factors responsible for longterm sick-leave, especially when there are no adjustments made before RTW, for example switching the department or not having to do certain challenging tasks or shifts anymore. Some GPs indicated that many of their patients with a MHD had problems in addressing that they are overwhelmed at work. Another crucial reason for the reluctance of RTW can be the sometimes justified feeling that employees could be penalized by their employers because of their disease and their requests of adjustments regarding the workplace or sickness absences associated with it.

“(…) I often witness situations of patients slipping into a depression at work because they had a somatic disease in the first place, which then led to them being off work for a prolonged time. For example, one patient broke a major bone playing a sports accident. He had to be off for half a year and when he returned, he was bullied massively by the corporate management. As a result of that treatment, he slipped into working disability and suffered burnout. And that takes its time, until they are on the road again” (I11:16).

“It is never the work by itself, rather the social environment at the workplace. Or that it is impossible to make the grade like the supervisors desire. Background: Consolidation of work” (I9:51).

On the other hand, GPs offered three main reasons why work remains fundamental for the recovery of their patients from MHDs: self-fulfillment, daily structure, and being in a social environment.

First, patients find so much more than just work at the workplace by interacting with their colleagues and using their skills to complete the tasks, they gain and maintain self-confidence and the feeling of fulfilling a purpose. Then, having to get up in the morning, being on time and having certain responsibilities there, gives their patients’ daily life more structure. Lastly, for some of their patients, the social network they find at the workplace is the only one they have. Thus, the workplace can be seen as a place to strengthen the resilience of patients with MHDs. When characterizing the workplace as positive, some GPs used a picture describing workplace as the solution to get patients gradually out of the depths of depression.

“(…) but then sometimes you feel cooped up in there. They sit at home, thoughts going round in circles and about work. Actually, then they realize that work is in fact not that bad, you know? That it is something that absolutely helps to making it out of a deep valley” (I7.33).

“(…) it is absolutely correct that work is very important for the patient. Yes, for self-confidence also. And that is something that is completely lost and may lead to the people falling into an even deeper hole. Yes, because there is simply something missing about the daily rhythm” (I5:35).

“And it depends on the life circumstances of a patient. If they live on their own, then they might feel secure at the workplace and can practice there” (I4:40).

Maybe this ambivalence towards work explains why GPs and patients themselves have a hard time in defining the right moment to RTW. It seems as if some patients can benefit from an early RTW.

“This is the question I ask my patients in the process of the treatment. (…) and I try to identify if work stabilizes or stresses them” (I9:51).

“So (…) with depression, you somehow reach a moment where you get a feeling like the valley bottom is crossed now. From my point of view. Some patients (laughs) must cross several valley bottoms” (I8:20).

“The longer the sick leave, the more difficult it gets, you know?” (I2:27).

### Optimization of the collaborative interfaces is beneficial for GPs and their patients

When treating patients with MHDs, several other professional and non-professional groups can be involved, besides GPs, which led us to answers regarding RQ5. In this study, the researchers had the overall impression that there is very little collaboration with other stakeholders, while having a good network is fundamental for the GPs. It saves them time, offers support, and helps with insecurities. Therefore, it can also speed up the process for the patients and thus may lead to a quicker successful RTW. As an example of problematic collaboration, GPs mentioned the increasing shortage of psychotherapy slots.

“Because if you think about it, when you are looking for a treatment place, you sometimes must wait 6 to 9 months until you get one. How is that supposed to work? This cannot work. But that is the crux of it. That there is somehow a therapeutic no man's land. Where nobody feels responsible” (I6: 45).

The GPs additionally indicated that the transition from psychiatric hospitals to outpatient treatment can often be problematic. For example, patients are discharged and labeled as fit for work when they actually are not. They are also left alone while rebuilding their daily routines at home. Finally, GPs explained that it makes a huge difference if employers are truly interested in getting their employees back after a long absence from work. They pointed out that a general understanding of MHDs by management at workplaces can aid a successful return. Concerning RQ6, GPs told us that the overall return system appears to be better established at larger employers. They emphasized on the value of gradual reintegration when RTW, as it is often used in Germany.

“Benevolent employers, who are not expecting full commitment and workload, and that is why I like it, that patients are still on a sick certificate. That would counteract the rehabilitation in my opinion. It is an accompaniment and an exploration.” (I4:35).

“Of course, we need a very sympathetic employer there. And at the workplace, we need some kind of care or friendly reception, you know? Accommodation, appreciation, especially with this problem. That is in my opinion something that can be an obstacle” (I2:65).

## Discussion

### Main findings

This study showed that GPs see themselves as very relevant in the rehabilitation of patients with MHDs because they can help with medical, administrative, and social issues, playing different roles for their patients, for example companion, therapist, social worker, et cetera. This may also indicate a lack of involvement of other non-professional groups, as it is necessary for GPs to regularly take on roles that society and the health care system do not seem to provide for patients with MHD. On the contrary, it underlines the importance of GPs for this cohort of patients and their rehabilitation process (RQ1).

GPs need a large body of knowledge and many different competencies, ranging from medical to administrative ones. A lot of their expertise is drawn from work experience, experienced anamneses and the special relationship to their patients as a GP (RQ3). Because of that, they develop individual strategies to support their patients with MHDs (RQ2).

However, GPs often have difficulties fulfilling their roles satisfactorily because of the lack of time, the frustration with having no simple solution, or bureaucratic obstacles (RQ4). Improving the interfaces for collaboration with other health professions might help, as the GPs reported poor connection and rare interactions with other stakeholders (RQ5).

We also discovered many additional individual factors associated with long-term absence from work, such as personality traits, fear of stigmatization and the interpretation of the workplace as negative or positive to the patients is also crucial to successful RTW (RQ4, RQ6).

### Contributions to existing literature

To our knowledge, this study is the first to have investigated factors of RTW among people with MHDs using *reflexive thematic analysis* and interviewing GPs in Germany. Once more it was confirmed that GPs play an important part in the treatment of MHDs, as already shown in various studies [30].

One main finding of our study is the large variety of roles a GP can play when treating a patient with a MHD with providing a list of every mentioned role by the interviewed GPs.

Other studies have indeed investigated on the roles of GPs before, but mostly regarding special diseases or peer groups without mentioning MHDs, for example in cancer prevention [31]. Also, our study could show the sources of knowledge and strategies GPs use when treating patients with MHDs.

We could confirm that GPs often have insecurities regarding the treatment of people with MHDs and often feel pressured when repeatedly asked to certify sick leave or prescribe medication [32]. Other studies even suggested that they feel unsure about their diagnoses in general [33].

GPs indicated that the likelihood of success when treating MHDs is often linked to their patients’ personality traits, which is supported by scientific reports [34, 35]. Other studies hinted that the severity of reported symptoms seems to be a prognostic factor concerning sick leave [36]. De Vries et al. could also show how the attitude of people with MHDs toward their disease can somehow predict their RTW success [36].

Some GPs told us about the fear of their patients of being stigmatized by society or at the workplace. Existing literature suggests that when feeling stigmatized as a result of their MHD, patients can hesitate for longer before even consulting a physician [37]. The effect of work on patients with MHDs was previously investigated in another qualitative interview study among 30 GPs, which also showed ambivalent opinions about work [38].

A key result of our study picking up this topic is, that a positive meaning of work to patients can have a strong influence on a successful RTW. A qualitative Danish study, even identified two groups of GPs, showing the influence of GPs opinions on work: One mainly thought that patients should only receive sick leave for a shorter period and act to encourage their patients to RTW as soon as possible. The other group tended to think that work exacerbates their patients’ condition; therefore, they thought they were taking this pressure off them when certifying sick leave, while taking no special action to promote the RTW process [39]. This study concluded that the overall understanding of work among GPs is rather positive, but it became clear during our interviews that GPs are often hesitant about determining right time for their patients to RTW. This is also a common problem among employees themselves [34]. High workload, lack of validation, or the feeling of having to do useless work or being overqualified can damage the mental health of employees [40]. In comparison, being unemployed can have similar effects on health and even lead to a shorter life expectancy, whereas a fulfilling job can lead to an overall higher quality of life [41].

### Limitations

This study has the following limitations. The sample size was rather small due to recruitment problems during the beginning of the COVID-19 pandemic. Accordingly, the presence of selection bias cannot be ruled out. However, data saturation was reached. Also there was a time limit set beforehand, although the authors do not believe it to be a disruptive factor, because most of the interviews found its natural end before the 60 min were elapsed. Moreover, the results of this study are grounded on the experiences and opinions of selected German GPs. They are of course heavily influenced by the way the German healthcare system works. Therefore, the findings of this study cannot to be considered as transferable to other healthcare systems.

It was not investigated if there is a difference when RTW or concerning stigmatization regarding the type of diagnosis in the spectrum of MHDs. Also, the important issue of gender differences in help seeking, getting diagnosed with an MHD, regarding stigmatization and RTW, was not brought up during the interviews.

### Implications for research and practice

The recently growing proportion of sick leaves associated with MHDs shows the importance of the issue of MHDs and RTW. This explains why further research on the topic is desperately needed. Our study could show that existing interfaces have to be improved. The different stakeholders in the field of psychiatric care should focus on better cooperation with GPs. First of all, GPs themselves are in need of better support. More training concerning patients with MHDs could be helpful. Cooperation between experienced psychiatrists and GPs could potentially lead to more scientifically well-founded decisions [42]. We would also like to highlight the problem that there is still some work to be done on the education of GPs concerning the issue of work issues and RTW.

A recent study developing a training for GPs regarding work-related stress factors unfortunately had no significant effect on improving the patients’ work-related self-efficacy or better recognition of work-related stress factors by the GPs [43]. Maybe it would be interesting to investigate the success of interventions and trainings taking place earlier in medical education.

Moreover, other stakeholders could take over some of the GPs’ many roles in the future. For example, social workers could help guiding the patients through the RTW process. Another idea is to foster the use of “practice nurses” who, for example can, offer regular appointments. They could, according to their education, offer patients and their families support in the assessment and treatment of MHDs [44]. There is a need to further explore the roles of a family physician from the perspective of their patients, society, and other health professionals. This may help to sharpen the role definition of a GP, with the potential to find members of non-professional and professional groups able to take on some of the roles, possibly leading to a reduction in role pressure for GPs. This could ultimately lead to more time and space for fulfilling the "original" role of the GP, and even allow for more effort in interdisciplinary collaboration.

Additionally, better cooperation between GPs and employers may lead to a higher success rate of RTW. The promotion of pre-vocational training or supported employment appears to have a lot of potential in this context [45]. A recent study implied that greater RTW success was achieved when using targeted RTW interventions and already starting them while the patients are still on sick leave or at a psychiatric clinic [46]. A study has just started at Hannover Medical School using an RTW program designed for patients on sick-leave due to MHDs. This program connects medical and psychotherapeutic support with interventions at the workplace and offering web-based post-rehabilitation support [47].

Employers could enhance their RTW rates by paying more attention to trained external consultants such as disability managers [48]. Then, strengthening of institutions such as integration services is needed to support both employers and employees.

Finally, programs providing education about MHDs at the workplace could reduce the stigma for the patients, resulting in greater success when RTW [49].

## Conclusion

This study was able to provide further insight about the experiences and opinions of GPs regarding the treatment of patients with MHDs with special focus on RTW. We concluded that they think of themselves as important in the RTW process of their patients. Despite having a small sample size, this study could also generate hypotheses about potential starting points for improving RTW, some of which could certainly also be useful in other healthcare systems outside Germany.

### Supplementary Information

**Additional file 1: **Interview guide.

## Data Availability

Data collected and analyzed during the current study are not publicly available because of their confidential nature. The datasets used and analyzed during this study are available from the corresponding author (Martina Geipel ge68cux@mytum.de) on reasonable request. The thesis of M.G with a collection of quotations (in German) will be available from the authors after approval by the Medical Faculty of the Technical University of Munich.

## Abbreviations

GP: General practitioner
MHD: Mental health disease
RTW: Return to- work
COREQ: Consolidated criteria for reporting qualitative research checklist
PHQ: Patient health questionnaire
RQ: Research question
n/a: No answer
