# Supplementary material for: Returning-to-work after mental health-associated sick leave: a qualitative interview study exploring the experiences of general practitioners in Germany
Source: BMC Prim Care. 2023 Dec 2;24:261. doi: 10.1186/s12875-023-02219-x (PMC10693165; doi:10.1186/s12875-023-02219-x)
Supplement: Supplementary file 1 — Additional file 1: Interview guide. [file 12875_2023_2219_MOESM1_ESM.pdf]

Interview guide

	Key question	Check questions	Precise questions	Maintenance/ control questions
I	**Which experience do you personally have with working patients having MHDs?**	How can the patients be characterized?<br><br>Surroundings of your patients?<br><br>Particularities in comparison to patients with somatic problems?<br><br>Common or rather rare situation for you?	Which diseases/diagnoses do your patients have?<br><br>To what extent play addictions a role?<br><br>How would you describe your role with your patients with MHDs?	Can you tell me some more about that?<br><br>Can you describe that a little further?<br><br>Can you give an example to that?
II	**If you decide to give a patient with a MHD a sick leave, how do you proceed?**	Describe the process when a patient with a MHD visits your practice!<br><br>Do you face problems when writing a sick leave for a patient with a MHD, too?<br><br>What else is important to the patient?<br><br>How is your usual proceeding after writing the sick leave?<br><br>How do you evaluate the right time for your patient to return to work?	Are there special approaches, e.g. tools or typical questions that you use which help you evaluate?<br><br>Do you have a certain scheme for follow-up appointments?<br><br>Are there goal agreements with your patients?	Can you describe your proceeding some more?<br><br>Can you think of additional examples to that?<br><br>Do you remember a certain case in particular?
III	**What is important for patients with long term sick leave and/or with the need to go to a psychiatric clinic, in your opinion?**	Do you see any differences when comparing them to patients with a short-term sick leave?<br><br>What is your experience with RTW of those patients?	How is your definition of a ‘short-term’ and a ‘long-term’ sick leave?<br><br>Describe your first contact to a patient after his/her discharge from psychiatric clinic!	Can you give an example for that?
IV	**In your reckoning, what is important for a successful RTW?**	What can you tell about the circumstances at the workplace of your patients, how do they influence the patients’ situation?<br><br>How important are the different stakeholders usually engaged in a RTW process?	Many would consider work as the root or the fuel to a MHD. Others would claim that work or RTW is a cruel part of the treatment of patients having a MHD. How do you feel about that?	

		How would you describe your level of integration with other stakeholders taking part in the RTW process?<br><br>Social workers at the clinic, doctors at the clinic, outpatient psychiatrists and psychotherapists, health insurance, RTW managers, company doctors?<br><br>Do you think there is potential for improvement at certain interfaces?<br><br>How would you describe the role of the social surroundings?<br><br>What would **you** wish for, to improve the process for **you**?	Which other factors do you consider as relevant for a successful RTW?<br><br>What could be changed/has to chance in your opinion to alleviate the RTW process?	Are there any more factors?<br>Can you name any other stakeholders who are important in your opinion?<br><br>Do you have a certain example to that?
**V**	**Is there anything you would like to add, did we miss a spot important to the issue?**		Do you like to go in further detail about a specific point we mentioned before?<br><br>Did I miss a spot completely which is crucial to you when speaking about this topic?	
